# SeedQuant: a deep learning-based tool for assessing stimulant and inhibitor activity on root parasitic seeds

**DOI:** 10.1093/plphys/kiab173

**Published:** 2021-04-15

**Authors:** Justine Braguy, Merey Ramazanova, Silvio Giancola, Muhammad Jamil, Boubacar A Kountche, Randa Zarban, Abrar Felemban, Jian You Wang, Pei-Yu Lin, Imran Haider, Matias Zurbriggen, Bernard Ghanem, Salim Al-Babili

**Affiliations:** 1 Division of Biological and Environmental Science and Engineering, the BioActives Lab, King Abdullah University of Science and Technology, Thuwal 23955-6900, Saudi Arabia; 2 Institute of Synthetic Biology and CEPLAS, University of Düsseldorf, Düsseldorf 40225, Germany; 3 Division of Computer, Electrical and Mathematical Science and Engineering, Image and Video Understanding Lab, King Abdullah University of Science and Technology, Thuwal 23955-6900, Saudi Arabia

## Abstract

Witchweeds (*Striga* spp.) and broomrapes (*Orobanchaceae* and *Phelipanche* spp.) are root parasitic plants that infest many crops in warm and temperate zones, causing enormous yield losses and endangering global food security. Seeds of these obligate parasites require rhizospheric, host-released stimulants to germinate, which opens up possibilities for controlling them by applying specific germination inhibitors or synthetic stimulants that induce lethal germination in the host’s absence. To determine their effect on germination, root exudates or synthetic stimulants/inhibitors are usually applied to parasitic seeds in in vitro bioassays, followed by assessment of germination ratios. Although these protocols are very sensitive, the germination recording process is laborious, representing a challenge for researchers and impeding high-throughput screens. Here, we developed an automatic seed census tool to count and discriminate germinated seeds (GS) from non-GS. We combined deep learning, a powerful data-driven framework that can accelerate the procedure and increase its accuracy, for object detection with computer vision latest development based on the Faster Region-based Convolutional Neural Network algorithm. Our method showed an accuracy of 94% in counting seeds of *Striga hermonthica* and reduced the required time from approximately 5 min to 5 s per image. Our proposed software, SeedQuant, will be of great help for seed germination bioassays and enable high-throughput screening for germination stimulants/inhibitors. SeedQuant is an open-source software that can be further trained to count different types of seeds for research purposes.

## Teaser

Software that efficiently and automatically assesses the germination rate of parasitic seeds in in vitro bioassays enables high-throughput screening for germination stimulants and inhibitors.

## Introduction

Root parasitic weeds, such as witchweeds (*Striga* spp.) and broomrapes (*Orobanchaceae* and *Phelipanche* spp.), are one of the major biological threats to the production of major agricultural food crops (Musselman et al., 2001; Tank et al., 2006; Parker, 2012; Pennisi, 2010; Rodenburg et al., 2016), as infestation by these obligate parasites causes yield losses ranging from a few percent to complete crop failure (Gressel et al., 2004; Ejeta, 2007; Atera et al., 2012). They jeopardize global agriculture due to their variety of hosts (Xie et al., 2010): witchweeds attack cereal crops in sub-Saharan Africa (Gressel et al., 2004; Parker, 2012), while broomrapes infest noncereal crops in Central Asia and the Mediterranean area (Joel et al., 2007; Parker, 2012).

Despite differences in their host specificity and evolution in diverse agroecological zones, they exhibit a common life cycle distributed between under and aboveground phases (Butler, 1995; Ejeta, 2007; Scholes and Press, 2008; Westwood et al., 2010). Their life cycle starts in the underground with seed germination that requires—in contrast to nonparasitic plants—chemical stimulants, mainly strigolactones (SL), released by host plants to establish symbiosis with arbuscular mycorrhizal fungi under nutrient-deprived conditions (Bouwmeester et al., 2003; Xie et al., 2010; Al-Babili and Bouwmeester, 2015; Lanfranco et al., 2018). Upon germination, parasite seedlings direct their radicle (the embryonic root of the weed) toward host roots and form a haustorium that grows to connect the parasite to its host, to deprive the host plant of vital resources including water, products of photosynthesis, and nutrients (Yoder, 1999; Paszkowski, 2006; Irving and Cameron, 2009; Yoneyama et al., 2010). This allows the parasites to grow, break the soil surface, and continue their above-ground development to reach maturity: a single parasitic plant can produce tens of thousands of tiny and highly viable seeds that return into the soil and supply an already huge seedbank in constant expansion (Ejeta, 2007; Jamil et al., 2012).

The control of parasitic weeds is a very difficult and challenging task, since (1) the infestation detection at early stages is nearly impossible, (2) parasitic weeds are naturally resilient (seed longevity), and (3) the extremely high number of produced seeds builds huge seed reservoirs in infested regions (Parker and Riches, 1993; Joel et al., 2007; Aly, 2012). A number of control measures have been employed—including cultural, agronomical, mechanical, and chemical approaches, applied either individually or in an integrated manner by combining several methods (Eplee and Norris, 1995; Haussmann, 2000; Aly, 2012)—and helped in mitigating the impact of root parasitic plants. However, they have not been effective enough to adequately address the problem of cumulated seed reservoirs in infested fields (Ejeta, 2007; Cardoso et al., 2011). Therefore, research has focused on developing strategies to eradicate or reduce these seed banks.

The application of synthetic germination stimulants (SL analogs) in the host’s absence is a promising approach to significantly reduce parasitic seed banks, as it leads to the death of germinating parasites, that is “suicidal germination” (Kgosi et al., 2012; Zwanenburg et al., 2016; KountcHe et al., 2019). Alternatively, there is a growing interest in further exploiting SL dependency to develop specific germination inhibitors. Such compounds should block SL perception of parasitic seeds but not of host plants, allowing their application in the presence of crops throughout the growing season (Nakamura and Asami, 2014; Holbrook-Smith et al., 2016; Yoneyama, 2016; Hameed et al., 2018).

The performance of SL analogs/inhibitors in inducing/inhibiting parasitic seed germination has been assessed mainly by direct application to parasitic seeds placed on petri dishes (Matusova et al., 2005). In this in vitro bioassay, preconditioned seeds are usually distributed and germinated in wells or on small glass fiber filter paper disks and let to germinate after the application of the target compound. The parasitic seed germination rate is recorded manually, counting germinated (seed showing a white-transparent protruded radicle through the dark seed coat) and nongerminated seeds (NGSs) using a binocular microscope (Jamil et al., 2011). Albeit being a standard procedure that yields hundreds of pictures every month for laboratories studying SL and related parasitic plants; the germination bioassay is laborious, tedious, time-consuming, and hardly manageable in high-throughput screening of large libraries for SL analogs or inhibitors.

The count of GS can be eased using staining solutions to increase the visibility of the colorless radicles (Long et al., 2008) or converted into absorbance signals by translating the germination rate into spectrophotometer-readable values (Pouvreau et al., 2013). Yet, they failed to reduce the workload, while keeping an experimentally relevant sample size. A recent combination of a binocular microscope mounted with a charge-coupled device (CCD) camera (Leica Microsystems), coupled with the protocol of Matusova et al. (2005), has allowed the first steps toward computer-aided image processing (Jamil et al., 2018). However, the success of this approach is limited, as the ponderous manual assessment of the germination rate had not been addressed. Thus, there is an urgent need to develop a high-throughput method for rapid and reliable recording of the germination rate in bioassays, which may be addressed using object detection and classification from a computer vision and deep learning perspective.

Deep learning is a subfield of artificial intelligence that has recently shown powerful capabilities to learn meaningful visual representations, allowing breakthroughs in the field of computer vision (Bengio et al., 2013). Provided that the algorithm is appropriately trained, deep learning methods can lead to an effective high-level identification, characterization, and abstraction of raw data or images. The rapid growth in the popularity of deep learning approaches was motivated by the release of large-scale datasets such as ImageNet (Deng et al., 2009) and Microsoft Common Objects in Context (MS COCO; Lin et al., 2014), gathering millions of images annotated with a diverse range of object categories. Its development has been supported by the increased accessibility of graphics processing units (GPUs), important architectural changes such as residual connections to solve for vanishing gradients (i.e. the possibility to train deeper and higher capacity models; He et al., 2016) and, finally, user-friendly frameworks such as the PyTorch and Tensorflow software libraries. The application of deep leaning in plant science helped to effectively detect GSs of several crop species, including chili (*Capsicum frutescens*), tomato (*Solanum lycopersicum*), pepper (*Capsicum annuum*), barley (*Hordeum vulgare*), maize (*Zea mays*), rice (*Oryza sativa*), and the parasitic plant *Striga hermonthica* (Nguyen et al., 2018; Chaivivatrakul, 2020; Colmer et al., 2020; Masteling et al., 2020).

In this work, we used the well-established algorithm Faster Region-based Convolutional Neural Network (Faster R-CNN; Ren et al., 2015) to develop an automated computer vision-based tool to detect, count, and record the number of GSs and NGSs from image datasets. Faster R-CNN has been recognized as a top-performing object detection algorithm with a high prediction accuracy (Huang et al., 2017). It has been successfully applied for a variety of downstream vision tasks (Fan et al., 2016; Hung and Carpenter, 2017; Jiang and Learned-Miller, 2017; Kang et al., 2017; Chen et al., 2018). Leveraging this object detection algorithm, we implemented a software that detects and localizes the parasitic seeds on images. We report a counting algorithm with 94% accuracy, which allows a single-click detection, recognition, and counting of seeds. We show the broad applicability of our algorithm in assessing the germination rate of different parasitic plant seeds. Finally, we provide a platform, SeedQuant (project website: https://braguyjm.github.io/SeedQuant2/), allowing researchers to simultaneously process large numbers of germination bioassay images without the need of important compute resources. SeedQuant can be used on Windows, MacOS, and Linux operating systems (to download SeedQuant: https://github.com/SilvioGiancola/maskrcnn-benchmark) for in vitro germination bioassay for parasitic seed detection and germination rate estimation.

## Results

### Faster R-CNN-based detection algorithm development

In computer vision, object detection and object classification have attracted the attention of the research community. Object detection algorithms localize objects of interest in images by estimating the smallest bounding box surrounding those objects. Recently, the development of a supervised deep learning algorithm called Faster R-CNN (Ren et al., 2015) allows to successfully “attend” to proposed regions, localize, and classify objects. Faster R-CNN is an efficient two-stage method consisting of a Region Proposal Network (RPN) and an object detector (R-CNN) that takes raw images as input and extracts a meaningful feature representation based on a Residual Network (ResNet) backbone. A list of anchors from the feature representation proposes candidate objects of interest (RPN stage), which are classified and regressed to the bounding boxes of the object of interest (Region of Interest [ROI] Pooling stage). In order to develop a high precision and efficient germinated and nongerminated parasitic seed detection algorithm, we integrated multiple feature representations (backbones) based on ResNet. Transferring well-trained classification models and using them as the backbone for object detection is a common approach called transfer learning. This learning can lead to detection algorithms with a meaningful semantic representation of an image, which can further be leveraged to understand and localize the position of objects. Such a backbone network acts as a feature extractor that helps to propose regions of interest and, subsequently, bounding boxes with classes for objects.

We adopted four backbone networks, namely, R-50-C4, R-50-Feature Pyramid Network (FPN), R-101-FPN, and ResNeXt-101-FPN with 50 or 101 convolution neural network (CNN) layers, according to their name. By using residual blocks to address the vanishing gradient problem in their design, the backbones can have a large number of sequential layers (deep network). This significantly increases the capacity of the network to learn more sophisticated and richer representations, and ultimately improves detection accuracy. The ResNeXt-101-FPN (Xie et al., 2017) is an enhanced version of ResNet, which performs a set of transformations (lower-dimensional embeddings) inside residual blocks, allowing the network to increase its accuracy while preserving the same number of parameters. Two of our ResNet backbones use the FPN (Lin et al., 2017), a component designed to detect objects at multiple scales, that boosts detection performance of Faster R-CNN for the challenging COCO benchmark (Lin et al., 2014). The details of the four adopted backbones are given as follows:

- R-50-C4 uses the first four residual blocks of convolutional layers of ResNet-50 (network with 50 residual layers). This is the original baseline in the Faster R-CNN paper.
- R-50-FPN uses the ResNet-50 model with the FPN as a feature extractor.
- R-101-FPN uses the ResNet-101 (network with 101 residual layers) model with the FPN.
- ResNeXt-101-FPN uses the ResNeXt network with 101 residual layers and FPN.

These four different backbones were trained, tested, and their results compared for detection of germinated and nongerminated parasitic seeds and counting performance. The integration of deeper models with larger numbers of parameters (i.e. larger capacities), such as R-101-FPN and ResNeXt-101-FPN, is expected to benefit significantly from the pretraining; hence, to boost the performance of the developed algorithm.

### Training Faster R-CNN

**Figure 1:**
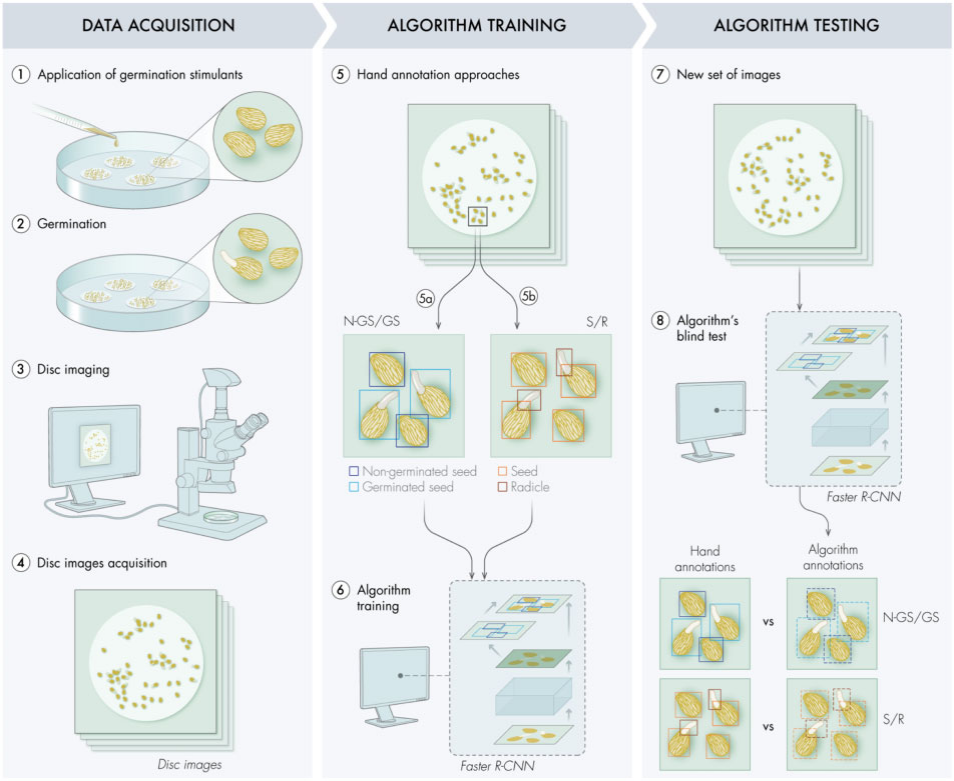
Pipeline for the algorithm development. Representation of the workflow to adapt an algorithm’s backbone for seed germination detection. During the data acquisition phase, germination stimulants were applied on preconditioned parasitic seeds (1), to trigger germination (2). Each disk, containing a mixture of GS and NGS, was placed under a binocular microscope mounted with a CCD camera for disk imaging (3–4). The acquired images were hand annotated (5) by placing tight bounding boxes around different objects following two classifications: (5a) NGSs/GSs and (5b) S/R. These annotated images were then analyzed by different Faster R-CNN backbones (6). Once the algorithm training is completed, a new set of images was provided to the different backbones for object detection (7). The algorithm annotations (NGS/GS and S/R), resulting from the blind test, were then compared with the hand annotations, performed simultaneously, to assess backbones performance (8).

The image dataset consisted of filter paper disks containing a mixture of germinated and nongerminated *Striga* seeds (Figure 1). To assess and obtain the most accurate object detection, we adopted and compared two annotation approaches: (1) NGSs versus GSs, where a tight bounding box is drawn to encompass either the seed alone (NGS) or the seed with its corresponding radicle (GS) and (2) S/R, where every seed is isolated from its respective radicle (if germinated). The first approach directly identifies germinated and NGSs as two different entities. However, for NGS/GS approach, GSs contain the seed element that can be recorded as an additional NGSs. Furthermore, radicles are usually overlapping, making the match between a seed and its radicle an arduous and useless task. The second approach tackles the problem in a semantic way by detecting all seeds (germinated or not) and all radicles separately, which largely reduces the possibility of false nongerminated detection.

The annotated input images with *Striga* seeds were used to train the four different backbones of Faster-RCNN: R-50-C4, R-50-FPN, R-101-FPN, and ResNeXt-101-FPN. For each model, we conducted two experiments where the parameters of the model were either initialized (1) with random weights by training the model from scratch using the “Xavier” weight initialization (Glorot and Bengio, 2010) or (2) with previous weights optimized for similar tasks (i.e. pretrained on an existing dataset MS-COCO (He et al., 2019)). As MS-COCO is generic with no seed class, we successively fine-tuned the parameters of the model on the seed detection task using the manually labeled image training set for *Striga* seeds. We optimized the numerous parameters of the model to output the expected detection results, following either NGS/GS or S/R approaches, to estimate the germination rate. We further augmented our dataset by artificially flipping the images around the vertical and horizontal axis.

**Figure 2:**
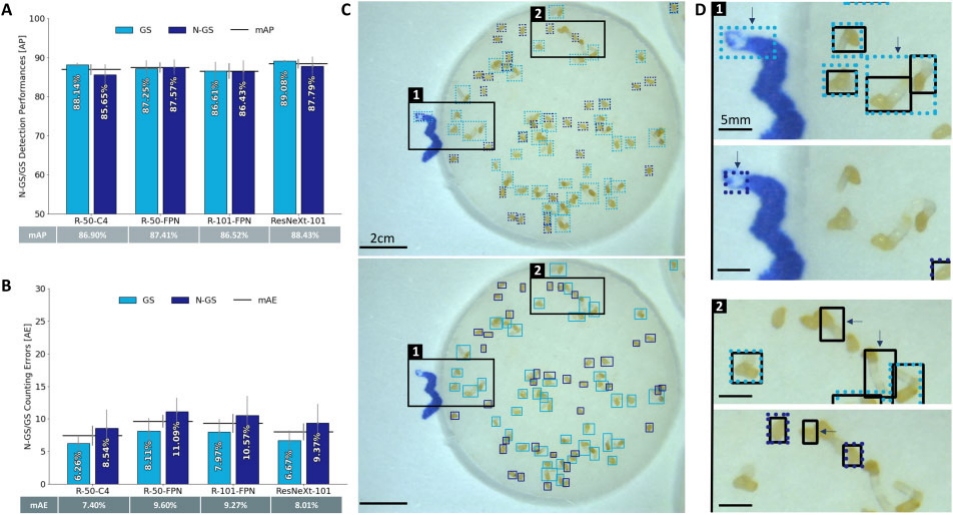
Performance assessment for NGSs/GSs classification on Striga seeds images. A, Detection performance on 32 images of the different Faster-CNN backbones (R-50-C4, R-50-FPN, R-101-FPN, ResNeXt-101) representing the accuracy (in percentage) of the predicted bounding box position in comparison to the hand annotated one (GT), estimated by the AP represented as bars with error bars that indicate standard error. The horizontal bar is representing the mAP for each backbone and its corresponding value can be found in the table below. B, Counting performance on 32 images of the different Faster-CNN backbones (R-50-C4, R-50-FPN, R-101-FPN, ResNeXt-101) in comparison to the hand counting of NGS/GS objects, estimated by the absolute error (AE). AE represents the percentage of count errors made by the different algorithms and is represented as bars with error bars that indicate standard error. The horizontal bar represents the mAE for each backbone and its corresponding value can be found in the table below. C, Visualization of the resulting bounding boxes for GS (light blue) and NGS (dark blue) on a disk image placed by (upper) the backbone R-50-C4 (algorithm prediction) versus (lower) the hand annotations (GT). D, Close up of the squares indicated on C, showing the variation of the annotation between GT (black bounding boxes) versus R-50-C4 prediction (GS in light blue, NGS in dark blue) on the same picture area.

**Figure 3:**
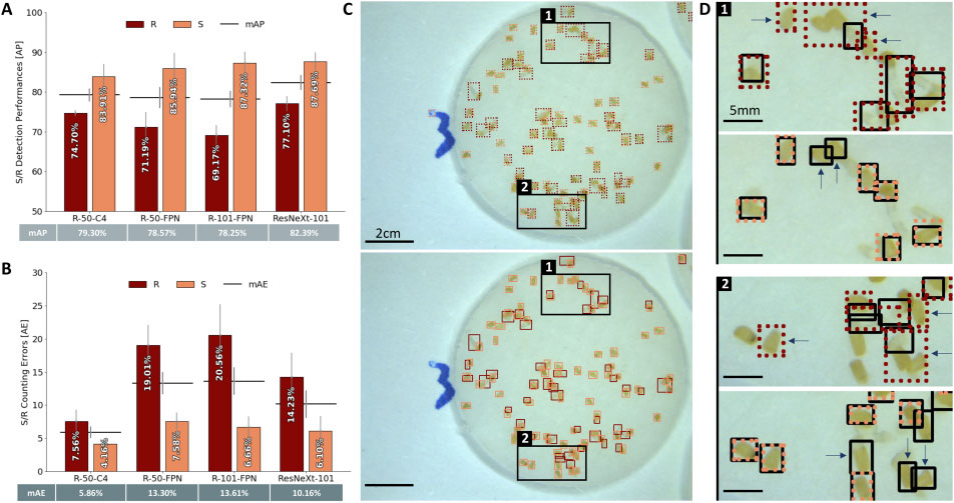
Performance assessment for S/R classification on Striga seeds images. A, Detection performance on 32 images of the different Faster-CNN backbones (R-50-C4, R-50-FPN, R-101-FPN, ResNeXt-101) representing the accuracy of the predicted bounding box position in comparison to the hand annotated one (GT), estimated by the AP in percentage represented as bars with error bars that indicate standard error. The horizontal bar represents the mAP for each backbone and its corresponding value can be found in the table below. B, Counting performance on 32 images of the different Faster-CNN backbones (R-50-C4, R-50-FPN, R-101-FPN, ResNeXt-101) in comparison to the hand counting of S/R objects, estimated by the absolute error (AE) percentage represented as bars with error bars that indicate standard error. The horizontal bar represents the mAE for each backbone. C, Visualization of the resulting bounding boxes for R (dark red) and S (light red) on a disk image placed by (upper) the backbone R-50-C4 (algorithm prediction) versus (lower) GT. D, Zoom in of the squares indicated on C, showing the variation of the annotation between GT (black bounding boxes) versus R-50-C4 prediction (R in dark red and S in light red) on the same area.

The eight different models (combination of four model architectures and two training strategies) were then validated by testing each model on images that were not trained on. Finally, object detection and recognition performance of the models were evaluated with a blind test: the algorithms predicted and placed bounding boxes on nonprocessed images according to the employed annotation approach. The resulting bounding boxes were then compared with the ones of the hand-made annotations (also called ground truth, GT), for both approaches NGS/GS and S/R. The comparison of the predicted bounding boxes against the GT was used to assess each model’s performances, for which two metrics were used: (1) the mean average precision (mAP), comparing the predicted position of the bounding box with the GT and (2) the mean absolute error (mAE) by comparing the count of each detected object class (NGS, GS, S, R) with the GT. As expected, the models pretrained on COCO produced results with slightly higher mAP and lower mAE (Supplemental Figure S1) compared with the models trained from scratch (Figures 2 and 3). The backbone architecture with the lowest mAE value (more accurate for counting) using one of the two annotation strategies (NGS/GS or S/R) was then chosen as SeedQuant’s backbone.

### Accuracy in GS and NGS detection

Realizing efficient and precise detection is essential for unbiased and reliable germination rate estimation. The NGS/GS classification resulted in consistently high detection performance among the different backbone architectures. The mAP values vary from 86.52% for R-101-FPN to 88.43% for ResNeXt-101 (Figure 2A). All backbones performed in a similar way, for both objects’ detection, with mAP values ranging from 85.65% for R-101-FPN N-GS to 89.08% for ResNeXt-101 GS. When compared separately, results revealed that ResNeXt-101-FPN and R-50-FPN are the most reliable backbones to efficiently detect both GS and NGS, with a gap of 1.29% and 0.32%, respectively.

On the other hand, the S/R annotation approach showed lower detection performance than the NGS/GS approach. The mAP values ranged from 78.25% for R-101-FPN to 82.39% for ResNeXt-101-FPN (Figure 3A). Moreover, the exact radicle detection on images was more challenging than the seed identification. Although seed-related average precision (AP) varied from 83.91% for R-50-C4 to 87.69% for ResNeXt-101, the radicle AP fluctuated from 69.17% for R-101-FPN up to 77.71% for ResNeXt-101-FPN, indicating a low accuracy for radicle detection.

For both annotation approaches, ResNeXt-101-FPN backbone displayed the highest mAP with 88.43% for NGS/GS and 82.39% for S/R (Figures 2, A and 3, A, respectively).

### Counting performance of the developed algorithm

As an important step for calculating the germination rate, the number of GSs and NGSs was counted for both annotation strategies. For the NGS/GS annotation strategy, results revealed similar counting accuracy for the different backbone architectures with a gap of 2.20% for the mAE values. The backbone R-50-C4 exhibited the most efficient and reliable counting, expressed by the lowest mAE value (7.40%) compared to 8.01% for ResNeXt-101-FPN, 9.27% for R-101-FPN and 9.60% for R-50-FPN (Figure 2B). For the NGS/GS annotation approach, NGS-related AE values are higher than GS AE values with a gap of 2.28% for R-50-C4 up to a gap of 2.98% for R-50-FPN.

On the contrary, S/R classification led to more disparate results across the different backbones with an mAE value ranging from 5.86% for R-50-C4 up to 13.61% for R-101-FPN (Figure 3B). Although the seed count was approximately similar (from 4.16% for R-50-C4 to 7.58% for R-50-FPN), the accuracy in the number of radicles was greatly variable with 7.56% for R-50-C4, up to 20.56% for R-101-FPN. This disparity comes from the radicle count, as each model is performing differently: 14.23% for ResNeXt-101, 19.01% for R-50-FPN, 20.56% for R-101-FPN while R-50-C4 is the most accurate with 7.56% AE.

Even though the NGS/GS annotation approach showed a better performance in mAP (accurate object detection) and lower mAE (number of individuals belonging to an object class) values than the S/R approach, the S/R based detection registered the lowest mAE value of 7.40% for N-GS/GS (R-50-C4) and 5.86% for S/R (R-50-C4). These results suggest that R-50-C4 architecture seems to be the most adapted backbone for discriminating germinated and nongerminated *Striga* seeds regardless of the annotation approach.

### Generalization on different parasitic seeds

We examined the knowledge transfer capability of the R-50-C4 backbone, combined with the S/R annotation approach, to assess whether the developed algorithm can be used on seeds of other root parasitic plants.

**Figure 4:**
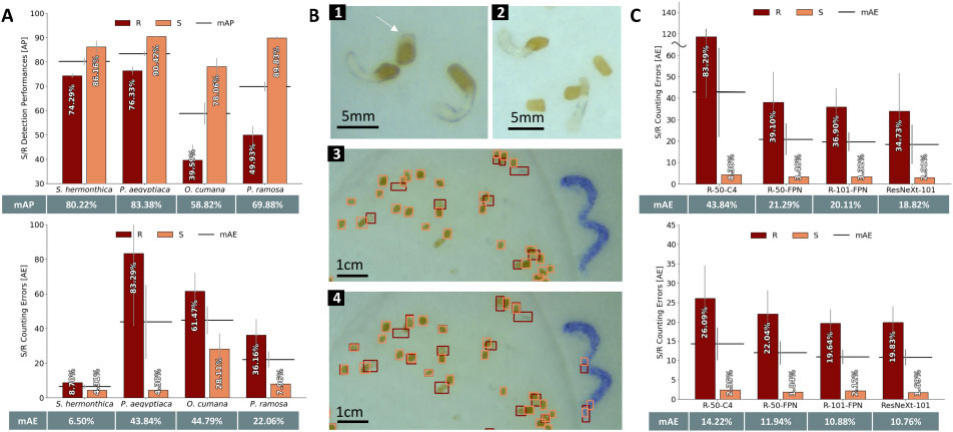
Generalization capabilities of the developed object detection algorithm for measuring germination of other parasitic seeds. A, Assessment of the performance of SeedQuant backbone (R-50-C4, developed on *Striga* seeds) on *O. ramosa, P. cumana*, and *P. aegyptiaca* seeds images (32 images for each seed type), in comparison with the hand annotations for the S/R (seed/radicle) annotation approach. Evaluation of (upper) the detection performances and (lower) the counting performances. The horizontal bars on the bar graphs represent the mAE/mAP, respectively, for each architecture and its corresponding value is reported in the table below. The error bars that indicate standard error. B, (1) Visualization of the *P. aegyptiaca* seeds morphology (nongerminated indicated by an arrow), showing their extra morphological structure, in comparison with (2) *Striga* seeds. Visualization of the bounding boxes placed by hand (3) and by the R-40-C4 algorithm (4) (seeds in light red, radicle in dark red). The direct processing of the *P. aegyptiaca* images by the *Striga*-based algorithm led to a lot of false positives in radicle detection, as the presence of the additional white part of the seed coat was classified as a very short radicle. C, Assessment of the counting performance of the different Faster-CNN backbone architectures (R-50-C4, R-50-FPN, R-101-FPN, ResNeXt-101) used for SeedQuant development on *P. aegyptiaca* seeds, based on the S/R object classification: (upper) prior fine-tuning on 32 images and (lower) after fine-tuning on 30 images, using 31 *P. aegyptiaca* seeds images for an additional training. The horizontal bars on the bar graphs represent the mean value of each architecture, its corresponding value is reported in the table below. The error bars that indicate standard error.

We used *Orobanche cumana*, *Phelipanche ramosa*, and *Phelipanche aegyptiaca* seeds, which are very similar to that of *Striga* (Supplemental Figure S2). We exposed them to similar experimental procedures, where each disk was individually photographed after 5–7 d of the exposure to a germination stimulant. We used the R-50-C4 S/R detection model from the *Striga* seeds training to predict bounding boxes on a set of 32 images of each seed type. The input image dataset was separately hand-annotated to assess the performances of the model on images of different parasitic seeds types and compared with the predicted annotations (Figure 4A;Supplemental Figure S3).

SeedQuant’s seed counting performance for *P. aegyptiaca* and *P. ramosa* was remarkable, with an error rate of 4.38% and 7.96%, respectively (Figure 4A). However, the seed counting of *O. cumana* was less efficient: 28.11% of the seeds were not detected by the algorithm. As expected, the detection and counting of radicles were more challenging, ranging from 36.16% for *P. ramosa* to 83.29% for *P. aegyptiaca*. The inefficient radicle detection, observed for *P. aegyptiaca*, corresponds to the presence of an inherent morphological characteristic of the *P. aegyptiaca* seeds, a small white protuberance as part of the seed coat, wrongly classified as radicle (Figure 4B(1), indicated by a white arrow).

To address the high error rate of *P. aegyptiaca* radicle counting, we further fine-tuned the R-50-C4 algorithm by performing an additional training using a set of 20 annotated images of *P. aegyptiaca* seeds followed by validation (11 images) and testing (30 images). As expected, the fine-tuning boosted both, mAP and mAE (from 83.29% down to 26.09% for radicles and 4.38% down to 2.35% for seeds; Figure 4C). We then used 20 newly annotated *P. aegyptiaca* images for training, 11 for validation and 30 for testing. All models reached between 20.58% and 23.02% error rate for radicle count and 1.61% up to 2.74% for seed count.

The protuberance of the *P. aegyptiaca* seed coat and the seed count error linked to *O. cumana* challenged the knowledge transfer of the model developed from *Striga* seeds to others seeds. This raised the question of the independence of SeedQuant toward the experimental settings. To address this question, we tested SeedQuant on collected disk images from two different research groups from the Netherlands Institute of Ecology (NIOO-KNAW), including the image dataset used to develop the *Striga* counting model DiSCount (Masteling et al., 2020), and Kenyatta University.

On a batch of images from NIOO-KNAW, SeedQuant conserved its performances with a mean counting error of 5.25% for seeds and 4.54% for radicles (Supplemental Figure S4A). However, SeedQuant showed a considerable error for radicle detection on DiSCount images (Masteling et al., 2020), due to the presence of plant debris (Supplemental Figure S4B). SeedQuant’s object detection was impaired on the images from the Kenyatta University as the number of seeds was undercounted, due to their relative darkness (Supplemental Figure S4C, upper part). Using a close up of the same image improved considerably the object detection; however, the dim light exposure affected the object classification and impeded the differentiation of seeds from radicles (Supplemental Figure S4C, lower part).

## Discussion

The development of a high-throughput tool for assessing the germination rate in parasitic seed germination bioassays is relevant for rapid characterization and screening of SL analogs or inhibitors, as well as the rapid identification and development of innovative alternative control strategies. This paper investigates efficient and high precision detection of germinated and nongerminated parasitic seeds using deep learning. Indeed, the reduced need for feature engineering, resulting in a significant gain of time and reliable quantitative data, is one of the most important advantages of using deep learning in image processing.

SeedQuant detects GSs using the S/R approach; a different strategy from the *Striga* seed counter DiSCount that relies on the radicle onset’s detection (Masteling et al., 2020). All our models performed counting very accurately; more particularly, models with larger capacities benefited from pretraining and generalized better. All backbones have AP values of at least 83.91% for seed detection, regardless of the annotation strategy, as seeds are generally of similar shape, size, and of contrasting color compared to the white background. However, the mAP values, assessing the average variation between predicted and GT bounding boxes (position and bounding box area) for each model, fluctuated between 78% and 90%. As predicted bounding boxes were sometimes placed loosely by the models, compared to the hand-annotated boxes (Figure 3D). This can be attributed to two characteristic elements of the input dataset including (1) the radicle attributes and (2) the high occurrence of object superposition.

When comparing the two annotation strategies, we observed a low accuracy for radicle detection from S/R classification (Figure 3). These white-transparent elements offer less contrast with the white filter paper (background) than the darker seed coats. Moreover, although subjected to the SL analogs at the same time, preconditioned parasitic seeds do not respond to these chemicals in a synchronized way. This results in the uneven development of radicles, growing into variable lengths. Their corresponding bounding boxes are less consistent in size and area than that of the seeds. Thus, it is possible that the radicle size variability and transparency have impeded their accurate identification. In addition, the presence of object superposition in the input images might have added another level of complexity in the object identification process. Most of the images contain an average of 60 elements (a mixture of GSs and NGSs), which oftentimes led to the superposition of tangled radicles on seed coats, hence complicating the detection for both types of object (Figure 3C, Box 2). Object superposition is also a challenge for hand annotation and can influence the accuracy of the GT as the bounding box placement on radicle knots can be misled by the intricacy of the intertwinement.

For object count, the absolute error (AE) values were generally low, with a maximum of 20.56% for radicle count using R-101-FPN. This corresponds to a minimum of 86.39% counting accuracy, with a maximum of 94.14% (mAE of 5.86%) using the R-50-C4 model, the least complex model. We speculated that more complex algorithms’ performances (with FPN) would have stood out if applied on a larger training set of images or for objects with different scales.

For SeedQuant development on *Striga* seeds, we prioritized accurate counting over exact detection, as a low AP value (above 65%) still allows detection of the object, thus, correct counting. The SeedQuant’s error rate of 5.86% is comparable to its counterparts for crop and *Striga* seeds (from 3% up to 13.27% error rate, Chaivivatrakul, 2020; Masteling et al., 2020), and better than the human error for large annotation tasks including hundreds of pictures—estimated at 5.85% during the annotation of the input images.

It is also worth noting R-50-C4’s performances can be further improved by conducting experiments on larger batches of images, as a larger training set is expected to further refine its learned detection parameters.

The direct generalization of SeedQuant on other parasitic seeds, such as *O. cumana*, *P. ramose*, and *P. aegyptiaca* seeds was impaired by a slight variation in seed color and morphology that misled the models. Although the seed coat detection was very accurate (4.38% and 7.96% error in seed count for *P. aegyptiaca* and *P. ramosa*, respectively, in opposition to 28.11% for *O. cumana*), the models were perplexed with the radicle detection. They reliably detected germinated radicles while reporting high-mAE values (from 36.16% up to 83.29%; Figure 4A), hinting at the presence of numerous false positives. This misidentification was particularly important for *P. aegyptiaca* due to its seed morphology. These seeds display an extra white-transparent cuticle-like feature, very similar to a small length radicle observed for freshly germinated *Striga* seeds. Learning that every white-transparent object should be a piece of radicle based on the *Striga* training, our models were unable to associate by themselves this additional feature as a part of the seed coat; creating many false positives for radicle detection (Figure 4B(4)). Thus, the knowledge transfer from *Striga* to other seeds was successful; however, not as straightforward as expected.

The refinement of the preexisting *Striga* models by using *P. aegyptiaca* images for an extra training phase (also called fine-tuning) yielded better results in terms of count accuracy, with an acceptable maximum mAE value of 14.22% (Figure 4C). It is worth noting that during this generalization, only 31 pictures were used for fine-tuning (training + validation), compared to 129 (97 + 32) used for *Striga.*

Moreover, the fine-tuned algorithm performance was very similar to that of the models’ trained independently (Figure 4C;Supplemental Figure S5, respectively), showing a possible versatility in quickly and easily adapt the potential of SeedQuant to new types of seed. Moreover, we open-source SeedQuant, both the interface for seed counting and the code to train further models on different sets of images. All models trained in this article are available on cloudlabeling.org, which allows researchers to detect and count seeds remotely, without the need of any extra computational resources.

The use of SeedQuant on pictures from different laboratories allowed to identify some of the limitations of the counting algorithm: (1) the cleanliness of the seeds (Supplemental Figure S4B); (2) the intensity of the microscope’s light source (Supplemental Figure S4C); (3) the radicle length (Figure S6); and (4) the dependency on color images (Supplemental Figure S7). (2) And (4) depend on the experimental set-up, which could be overcome by the creation of new models using images from different laboratories. using we were able to provide some guidance to improve the first and third points. We detailed the handling of parasitic seeds prior germination (see “Parasitic seed cleaning”) to reduce the amount of plant debris in the seed batch, and illustrated a protocol to successfully perform bioassays while ensuring an appropriate radicle length (Supplemental Figure S8). Alternatively, the use of DiSCount for unclean seed batch is recommended, as the training image set contains plant debris (Masteling et al., 2020).

## Conclusion

In this study, we developed and validated a software, SeedQuant, as a high-throughput and efficient tool for parasitic seed detection and counting.

We adapted the robust object detector algorithm Faster R-CNN, using Deep Neural Networks-based transfer learning, and developed an algorithm for germinated and nongerminated parasitic seed detection. This algorithm, established from a trained version of R-50-C4 backbone architecture, was capable of accurately assessing (up to 94% counting accuracy) the rate of parasitic seed germination from image datasets. We demonstrated the possibility of knowledge transfer from *Striga* spp. to other parasite plant families, such as *Orobanche* and *Phelipanche* spp., although we recommend species-specific fine-tuning for improved performances.

**Figure 5:**
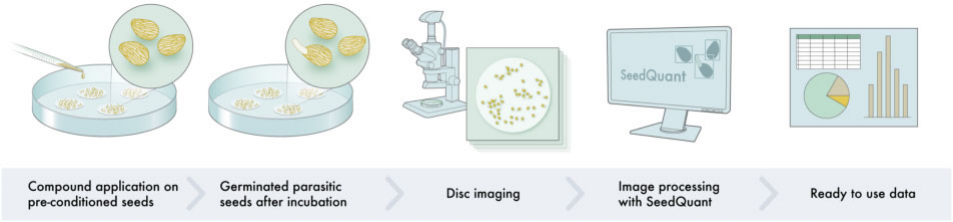
Proposed workflow for seed germination stimulants/inhibitors studies using SeedQuant. Stimulants or inhibitors are applied to preconditioned seeds, placed on small (9 mm) glass fiber filter paper disks. After incubation, the disks are imaged under a binocular microscope mounted by a charge-coupled device (CCD) camera. In one click, the resulting pictures are processed by SeedQuant, which renders the germination ratio for each picture. The data are then provided to the user in an easy-to-use format (.csv), allowing further data processing.

We packaged the developed seed detection model into SeedQuant, a fast, reliable, and high-throughput software for parasitic seed detection and counting from in vitro bioassay’s image dataset. SeedQuant leverages the boresome and routine work of counting parasitic seed germination rate without the need of important compute resources, but limited to an internet connection. It is open source and can be downloaded at https://github.com/SilvioGiancola/maskrcnn-benchmark. This software has a user-friendly interface that allows the almost-instantaneous processing and characterization of a single/group of image(s) into an exportable table, which logs image name and germination rate information (Figure 5). The latter summarizes the count details, for all images, in an open CSV (comma-separated values) format and enables further statistical per-image analyses, as well as per-batch analysis. Additional data can be retrieved from the processed pictures by slightly modifying the code and using visual instance segmentation techniques. As a matter of fact, each bounding box contains much information (pixel color, bounding box size), which could be useful for further characterization of germination stimulants and inhibitors.

## Materials and methods

### Biological data

#### Parasitic seed material

*Striga hermonthica* seeds were collected from sorghum fields during the 2012 rainy season in Sudan and were generously provided by Prof. Abdel Gabbar Babiker. Seeds of *Phelipanche aegyptiaca*, collected from Egypt, were kindly provided by Dr Mohamed Abdul-Aziz, University of Cairo. *Phelipanche ramosa* seeds were kindly provided by Philippe Semier, Université de Nantes, France, and *O. cumana* seeds were generously provided by Dr Alberto Martin Sanz from the University of Seville, Spain.

#### Parasitic seed cleaning

For fine cleaning, seeds were soaked in water for 30 min. Meanwhile, 15 mL of 60% (w/v) sucrose solution was added in a 50-mL tube, followed by 10 mL of 40% sucrose added dropwise to create concentration gradient. About 20-mL water, along with *Striga* seeds, was then added dropwise to the 50-mL tube and centrifuged for 10 min. The middle layer of the seeds was collected gently, washed thoroughly and dried in a laminar fume hood.

#### Preconditioning of parasitic seeds

Parasitic seeds require specific temperature and moisture conditions for a certain period of time (preconditioning) to germinate. For this purpose, *Striga* seeds were surface-sterilized in a 50-mL tube with 20% bleach with 0.1% Tween-20 for 5 min. For *Phelipanche/Orobanche*, seeds were surface-sterilized in a 50-mL tube with 75% ethanol for 3 min. After removing the bleach/ethanol with subsequent six washings, the seeds were again sterilized with 3% bleach for 3 min, poured along with bleach, were poured in vacuum assembly (Sigma Aldrich) placed under a laminar flow cabinet, and thoroughly rinsed with six subsequent washings with sterilized milliQ water. The seeds were shifted in a glass petri plate and air-dried under a laminar flow cabinet for 3–4 h. Approximately 9-mm glass fiber disks were put in a glass petri plate and parasitic seeds (∼50–100) were spread uniformly on each disk. Then 12 disks were carefully transferred on a sterilized 90 mm Whatman filter paper (Merck KGaA, Darmstadt, Germany), placed in a plastic petri plate and moistened with 3-mL sterilized milliQ water. The plates were sealed with parafilm (Merck KGaA, Darmstadt, Germany) and covered in an aluminum foil. The plates with *Striga* seeds were put in an incubator at 30°C for 10 d, while the ones containing *Phelipanche*/*Orobanche* seeds were incubated at 21°C for 14 d.

#### Germination of parasitic seeds

Following preconditioning, the plates were opened and air-dried under a laminar flow cabinet. For *Striga*, a new labeled plastic petri plate was supplemented with a 90 mm Whatman filter paper ring, supplied with 900-µL sterilized milliQ water, and six dried disks containing preconditioned seeds. A 55-µL of germination stimulant was added on each disk. For *Phelipanche*/*Orobanche*, six dried disks containing preconditioned seeds were transferred into a 24-well plate and 100 µL of germination stimulant was added on each disk. Plates were sealed with parafilm (Merck KGaA, Darmstadt, Germany) and incubated at 30°C for *Striga* (24 h) or 25°C for *Phelipanche*/*Orobanche* (5 d). A protocol describing the critical steps is depicted in Supplemental Figure S8.

### Dataset acquisition

#### Imaging of parasitic seed disks

After germination, the Petri plates were taken out from the incubator, opened and air-dried in ambient conditions for few minutes to get rid of extra moisture. Each disk, containing a mix of 50–100 germinated (exhibiting a translucent radicle poking out of the dark seed) and NGS (seed alone), with an average germination of 55% per disk, was individually photographed using a Leica LED3000 R adjusted to 50% medium light, mounted with a CCD camera (Leica Microsystems). The images were saved in 8-bit, 2,592 × 1,944 pixels and exported in JPEG format using the LAS-EZ-V3-0 software for image acquisition.

#### Building a dataset for seed Ccnsus

A set of 161 images were produced with a total of 9,826 *Striga* seeds. We used 97 images (∼60%) to train our deep learning algorithms and 32 images (∼20%) for validation to avoid overfitting our models to the training (i.e. memorizing the training images). We kept 32 extra images (∼20%), previously unseen by the trained model, to perform a blind test. For *P. aegyptiaca*, 61 images containing a total of 3,529 seeds were used: 20 for fine-tuning, 11 for validation, and 30 for the blind test of our models.

The image annotation followed two logical approaches to detect the seeds (germinated or not) and identify their germination ratio: NGSs/GSs detection and S/R detection. This annotation process consisted of drawing tight bounding boxes around the objects of interest using the software LabelImg (Tzutalin, 2015), indicating the coordinates and the class of the object encapsulated inside each bounding box: they are considered as GT, that is, the expected output of our algorithm. Resulting annotations were exported in XML files following the Pascal VOC format (Everingham et al., 2010), one of the most commonly used annotation formats for object detection tasks. Our dataset—gathering images and annotation files—is scalable, and, as any other deep learning algorithm, our model would benefit from further annotated images.

### Algorithm details

#### Feature representation

In our experiments, we adopted four backbone networks, namely, R-50-C4, R-50-FPN, R-101-FPN, and ResNeXt-101-FPN. The first three backbones are variations of the popular ResNet network (He et al., 2016) with either 50 or 101 layers of CNN.

#### Region proposal network

The features (feature maps) extracted with a backbone are further used to generate object proposals. For each pixel of the feature maps, we proposed nine anchors, that is, nine bounding boxes centered at each location of the feature map, with three different scales and three different aspect ratios. Those nine different anchors would help in coping with various shapes of objects. Each anchor is binary classified based on an objectness, that is, whether it represents an object or a background. We regressed four scalars for each positive anchor to adjust the bounding box to be tight around the object it contains. In total, two classification and four regression values are estimated per anchor. We defined positive and negative anchors based using their Intersection over Union (IoU) with the GT bounding boxes. In particular, anchors with IoU >0.7 were defined as positive, while anchors with IoU <0.3 as negative. The anchors were further classified using a cross-entropy loss and the position of the positive boxes regressed to their assigned GT using a smooth L1 loss.

#### ROI Pooling

We successively pooled and cropped the feature maps around all positive bounding boxes regressed from the RPN stage. The features from the bounding boxes are successively elaborated with further CNN layers, in order to estimate its class and regress tighter bounding boxes. Similarly, to the RPN, the Fast R-CNN part has two branches (also called heads): regression and classification. Note that RPN is class-agnostic, meaning it does not predict the object classes. In the detection stage, we predict scores for each class and an additional background class, for negative anchors. We normalized the classification scores (to sum up to 1) and to represent the probability distribution for object classes using a softmax over the final layer prediction. Similar to the RPN, the classification is learned with a cross-entropy loss and the regression with a smooth L1 loss. During inference, we predict the class with the highest score. This value is referred to as the confidence score and can be used to threshold or evaluate the predictions.

#### Model training

Each suggested *Striga* model was trained for 5,000 iterations with batches of two images using the Adam optimizer with a learning rate of 0.01. The *P. aegyptiaca* models were trained for only 2,000 iterations as they contain less images. We trained all Faster-RCNN models with the four different backbones and retained the models at the iteration performing best on the validation set, in order to avoid overfitting on the training set of images. All models were first pretrained on the COCO dataset, in order to transfer visual knowledge learned from large-scale generic datasets.

#### Algorithm performance metrics

The trained Faster R-CNN models were evaluated on the blind test set of images defined previously. We evaluated the performance for both detection and counting.

#### Detection metric

The most common metric used to evaluate the performance of object detection is the mAP, which includes three parameters such as the IoU, precision, and recall. The IoU defines the tightness of a predicted bounding box with respect to the annotated one. In particular, the ratio between the intersection and the union of those two bounding boxes (annotated versus predicted) is used to decide whether a prediction is positive or negative. Setting a higher IoU threshold would restrict the performances for tight bounding boxes, while a lower IoU threshold would still consider looser bounding boxes as positive. On the basis of application, one can decide whether tight bounding boxes are required or not. Predictions are then classified as follow:

- True positive (TP)—predicted box has an IoU with a GT box that is higher than the threshold value.
- False positive (FP)—predicted box has an IoU with a GT box that is lower than the threshold value.
- False negatives (FN)—no predicted box for an object.

On the basis of those definitions, the precision is estimated as the ratio TP/(TP + FP) and represents the ratio of predicted correct objects. Also, the recall is estimated as the ratio TP/(TP + FN), and represents the ratio of objects that the tested model missed to predict. In addition, each prediction is provided with a confidence score, it is at discretion of the user to consider predictions above a given confidence score. Focusing on high-confidence scores would improve the precision of the method, but worsen the recall as many instances would be missed. Similarly, considering lower confidence scores would improve the recall but worsen the precision as many false detections would appear. As a tradeoff between precision and recall, the AP metrics is commonly used as the area under the Precision–Recall curve drawn with all possible combinations of precision/recall. As this metric is defined for a single class of object, the mAP provides the mean of the AP for each class of object of interest (NGS/GS or S/R).

#### Counting metric

Since our final goal is counting, we also evaluate the mAE as follows:

$$\mathrm{mAE}=\frac{1}{N}\sum_{i\;=\;1}^{N}\frac{\mathrm{abs}(y_{i}-t_{i})}{y_{i}}$$

where *N* is the number of classes, *y_i_* the GT count for the object class *i*, and *t_i_* the predicted count for the object class *i*.

We will show below that while one could be tempted to calculate solely the standard detection metric (mAP), it is essential to do counting metrics analysis as in general, a model producing results with higher mAP does not result in the smaller counting error.

#### Inference speed

We recorded and analyzed the detection speed—how much time does it take for a network to produce an output when it is presented with an input (raw image)? We estimated that a scientist takes on average 5 min to count a disk and compared it to the different backbones (Table 1).

**Table 1:** Inference time of the different backbones for SeedQuant development

Backbones	R-40-C4	R-50-RPN	R-101-RPN	ResNeXt-101-FPN
Inference Speed (s)	0.241^a^	0.086	0.102	0.173

All inference times were obtained using an NVIDIA GTX1080Ti GPU.

^a^ Our method is *1200x (R-50-C4) to 3600x faster (R-50-RPN)* than an experienced researcher for this counting task

### Statistical analysis

We trained each model for 30 times, using different random initializations for the weights. Then, we predicted the bounding boxes on the blind test set of images, counted the object of interest (NGS/GS or S/R) and reported the per-class and average results for each architecture.

## Supplemental data

The following materials are available in the online version of this article.

**Supplemental Figure S1.** Performance assessment of the four different model architectures for both annotation approaches (NGS/GS and S/R), pretrained on COCO, for *Striga* seeds.

**Supplemental Figure S2.** Image acquisition of the different parasitic seeds used for the study.

**Supplemental Figure S3.** SeedQuant’s knowledge transfer to *P. ramosa* and *O. cumana*.

**Supplemental Figure S4.** SeedQuant’s independence test on images containing *Striga* seeds.

**Supplemental Figure S5.** Model performance evaluation of training from scratch on *P. aegyptiaca* seeds for both annotation approaches (NGS/GS and S/R).

**Supplemental Figure S6.** R-50-C4 backbone test on long radicles-containing images with *Striga* seeds.

**Supplemental Figure S7.** R-50-C4 backbone test on grayscale images.

**Supplemental Figure S8.** Illustrated step-by-step protocol for parasitic seeds bioassay.

## Supplementary Material

kiab173_Supplementary_Data

## Notes

J.B., M.R., S.G., B.A.K., and M.J. developed the project and wrote the article. M.R. and S.G. adapted, trained, and created different algorithms. J.B., B.A.K., and M.J. were responsible for the biological part of this work: image collections and annotations. M.R. and S.G. developed the user interface with the inputs from J.B. J.B, B.A.K., M.J., R.Z., A.F., J.W., P.L., and I.H. were involved in the data collection by placing bounding boxes around the different objects to identify. M.Z. contributed to the discussion and planning of the project. S.A.B. and B.G. initiated and supervised the project. S.A.B. and B.G. agreed to serve as the authors responsible for contact and ensure communication.

The author responsible for distribution of materials integral to the findings presented in this article in accordance with the policy described in the Instructions for Authors (https://academic.oup.com/plphys/pages/general-instructions) is: Salim Al-Babili (salim.babili@kaust.edu.sa).
