# Supplementary material for: SeedQuant: a deep learning-based tool for assessing stimulant and inhibitor activity on root parasitic seeds
Source: Plant Physiol. 2021 Apr 15;186(3):1632–44. doi: 10.1093/plphys/kiab173 (PMC8260127; doi:10.1093/plphys/kiab173)
Supplement: kiab173_Supplementary_Data [file kiab173_supplementary_data.pdf]

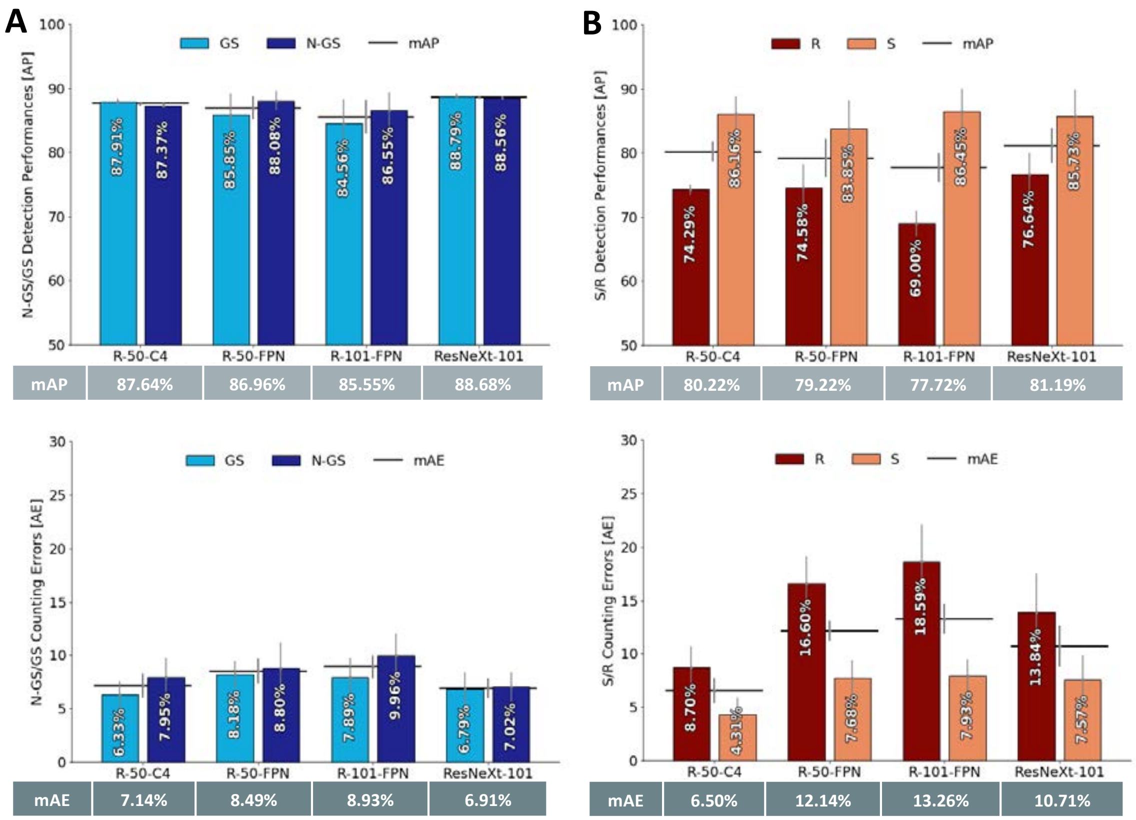

Supplemental Figure S1: Performance assessment of the 4 different model architectures for both annotation approaches (N-GS/GS and S/R), pre-trained on COCO, for *Striga* seeds. The backbones R-50-C4, R-50-FPN, R-101-FPN and ResNeXt-101 were first pre-trained on object detection using a large collection of images before being trained to recognize germinated and non-germinated seeds. The hand annotations, also called ground truth (GT) were compared with the predicted annotation for **A**- the non-germinated seed *vs* germinated seed (N-GS/GS) annotation approach, **B**- the seed *vs* radicle (S/R) object classification for *Striga* seeds. Each approach was evaluated using (upper graphs) detection performances, assessing the predicted bounding boxes position compared to the GT ones and (lower graphs) counting errors.

The horizontal bars on the bar graphs represent the mean value of each performance (mAP/mAE) for each architecture, and its corresponding value is reported in the table below.

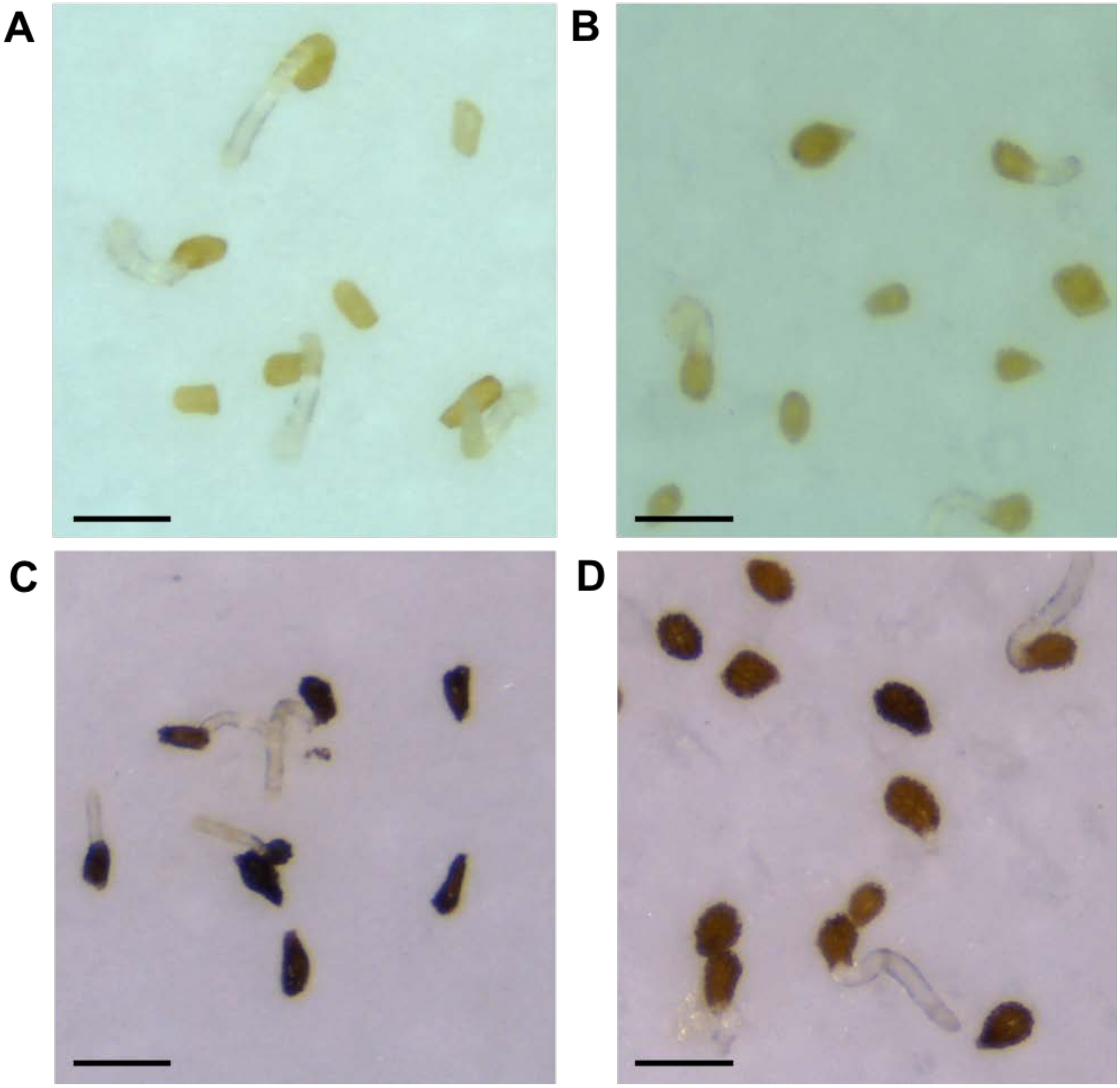

Supplemental Figure S2: Image acquisition of the different parasitic seeds used for the study: **A**- *Striga hermonthica*, **B**- *Phelipanche aegyptiaca*, **C**- *Orobanche cumana,* **D**- *Phelipanche ramosa*. All scale bars represent 5 mm.

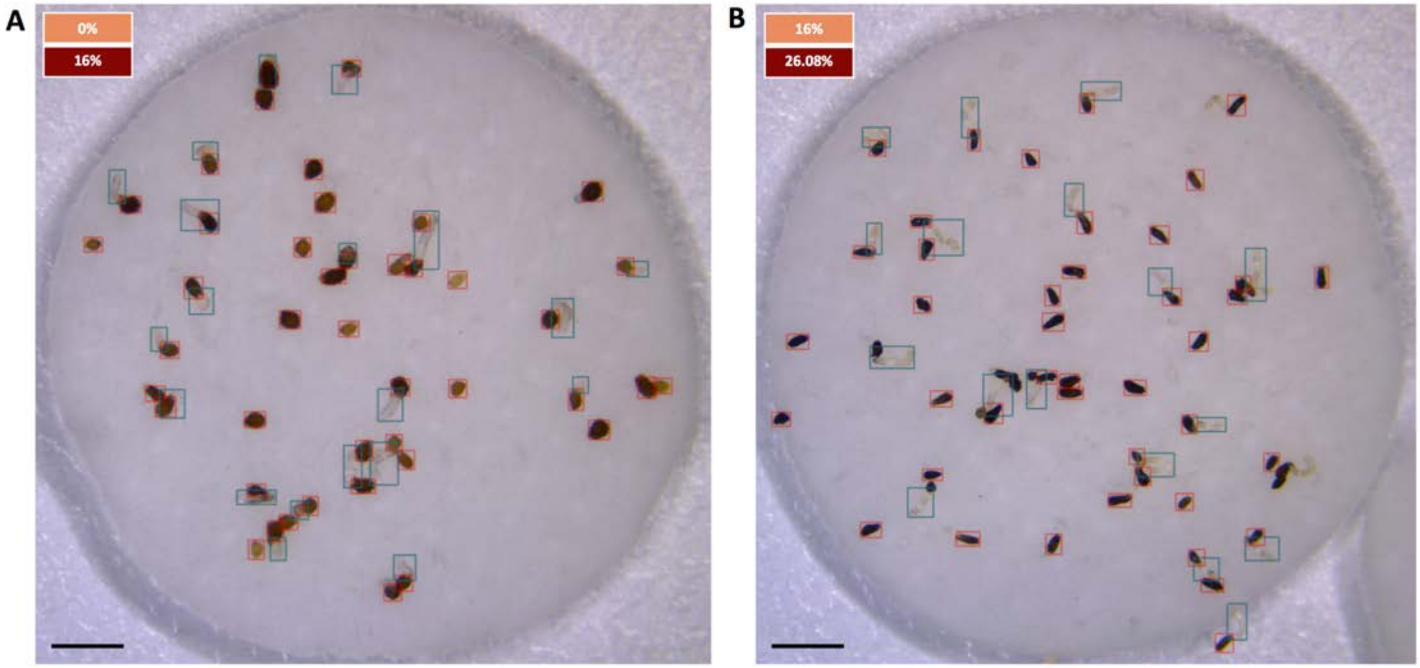

Supplemental Figure S3: SeedQuant's knowledge transfer to *P. ramosa* and *O. cumana*. Assessment of the counting performances of the R-50-C4 backbone following the S/R annotation approach using different parasitic seed species: **A-** *P. ramosa* and **B-** *O. cumana*. The slight morphological differences of the seed coat and radicle influenced a bit the detection performances of SeedQuant.

The error percentage for each image is reported in the upper left-hand corner: for the counting of seeds (light red) and radicles (dark red) between the R-50-C4 backbone and the ground truth. The detected seeds are indicated by red bounding boxes, and the radicles by green bounding boxes. The scale bars represent 1cm.

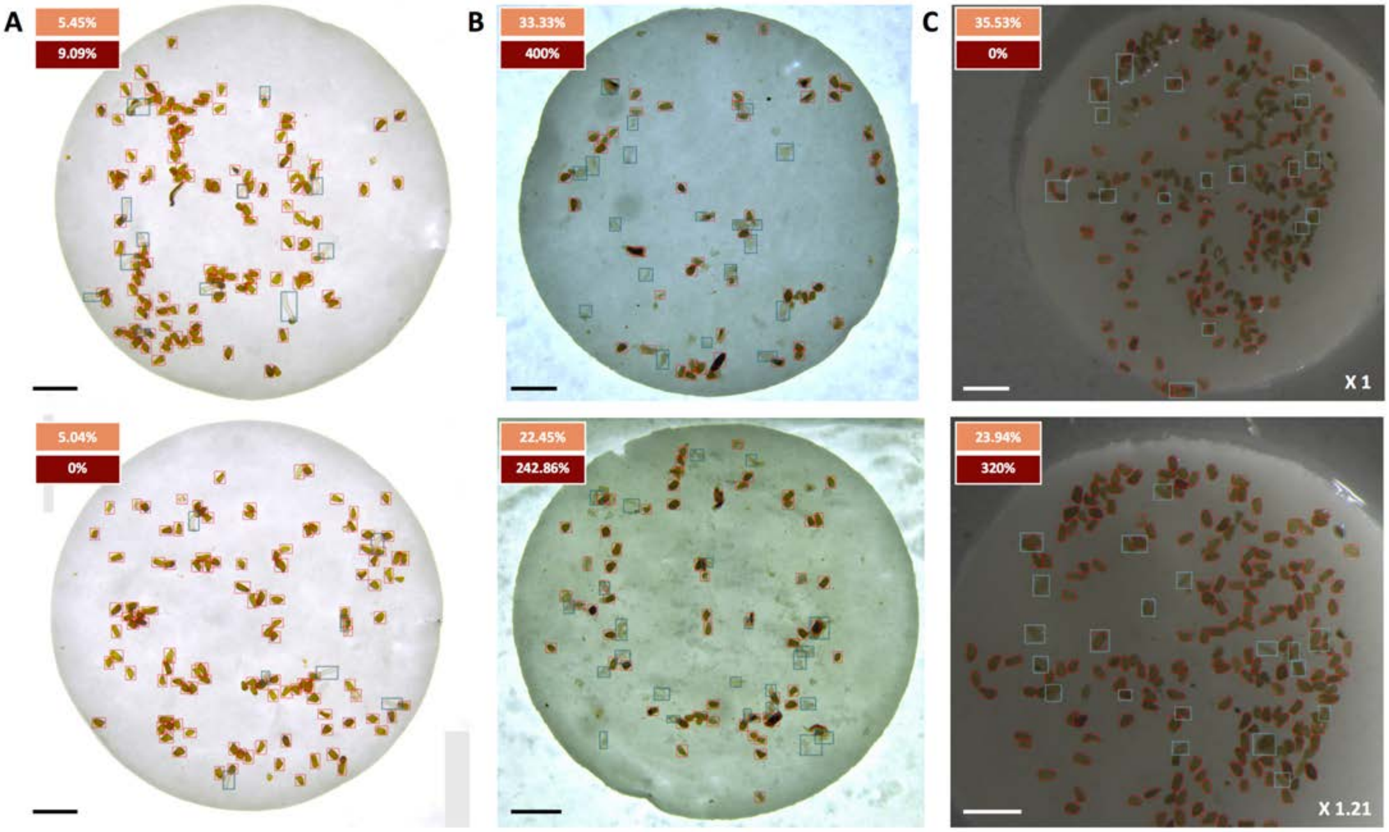

Supplemental Figure S4: SeedQuant's independence test on images containing *Striga* seeds. Assessment of the counting performances of the R-50-C4 backbone following the S/R annotation approach using images provided by two research groups: **A**, **B-** from the Netherlands Institute of Ecology in Wageningen (the Netherlands) and **C-** from the Kenyatta University in Nairobi (Kenya). The latter shows two magnifications of the same disc: x1 (up) and a close up (x1.21, down).

The error percentage for each image is reported in the upper left-hand corner: for the counting of seeds (light red) and radicles (dark red) between the R-50-C4 backbone and the ground truth. The detected seeds are indicated by red bounding boxes, and the radicles by green bounding boxes. The scale bars represent 1cm.

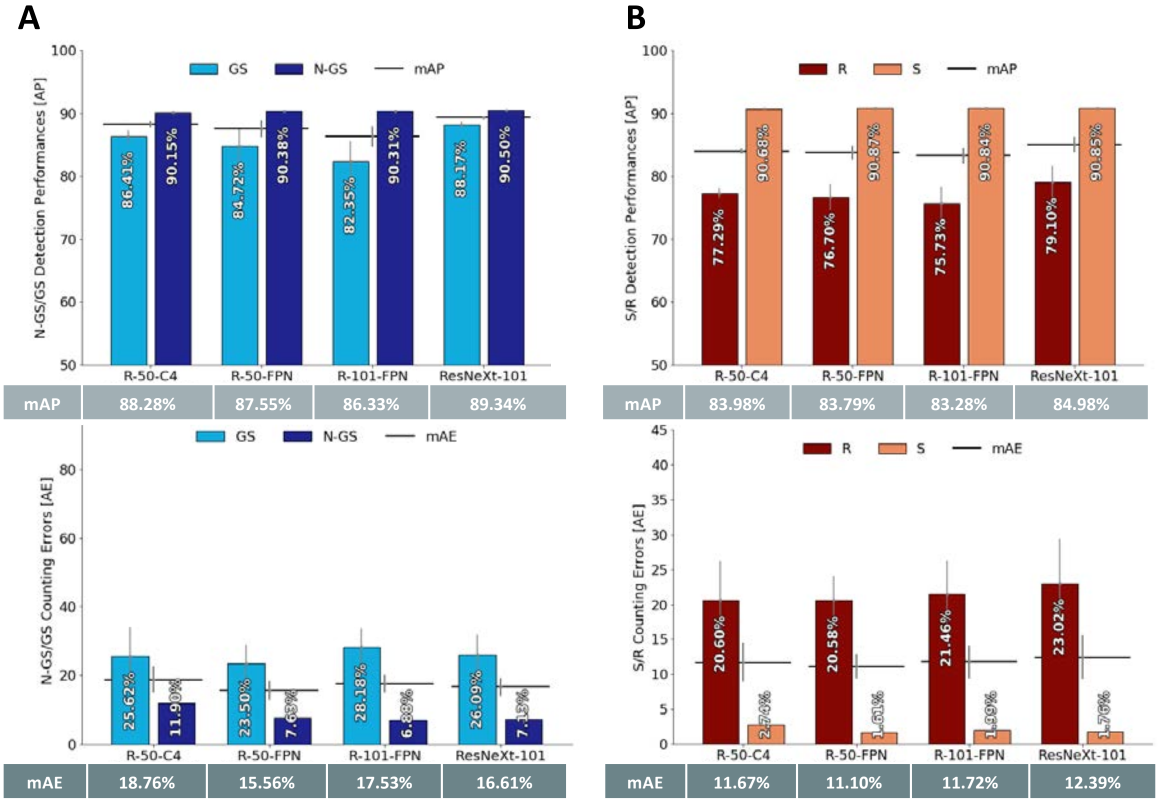

Supplemental Figure S5: Model performance evaluation of training from scratch on *P. aegyptiaca* seeds for both annotation approaches (N-GS/GS and S/R). The different Faster-CNN backbone architectures (R-50-C4, R-50-FPN, R-101-FPN, ResNeXt-101) were trained from scratch using only 20 *P. aegyptiaca* seeds images, creating a *Striga*-independent trained model. The predicted bounding boxes from these new algorithms were compared with the hand annotations for **A**- the non-germinated seed *vs* germinated seed (N-GS/GS) annotation approach, **B**- the seed *vs* radicle (S/R) object classification. Each approach was evaluated using (upper graphs) detection performances, assessing the predicted bounding boxes position compared to the GT ones and (down graphs) counting errors.

The horizontal bars on the bar graphs represent the mean value of each performance (mAP/mAE) for each architecture, its corresponding value is reported in the table below.

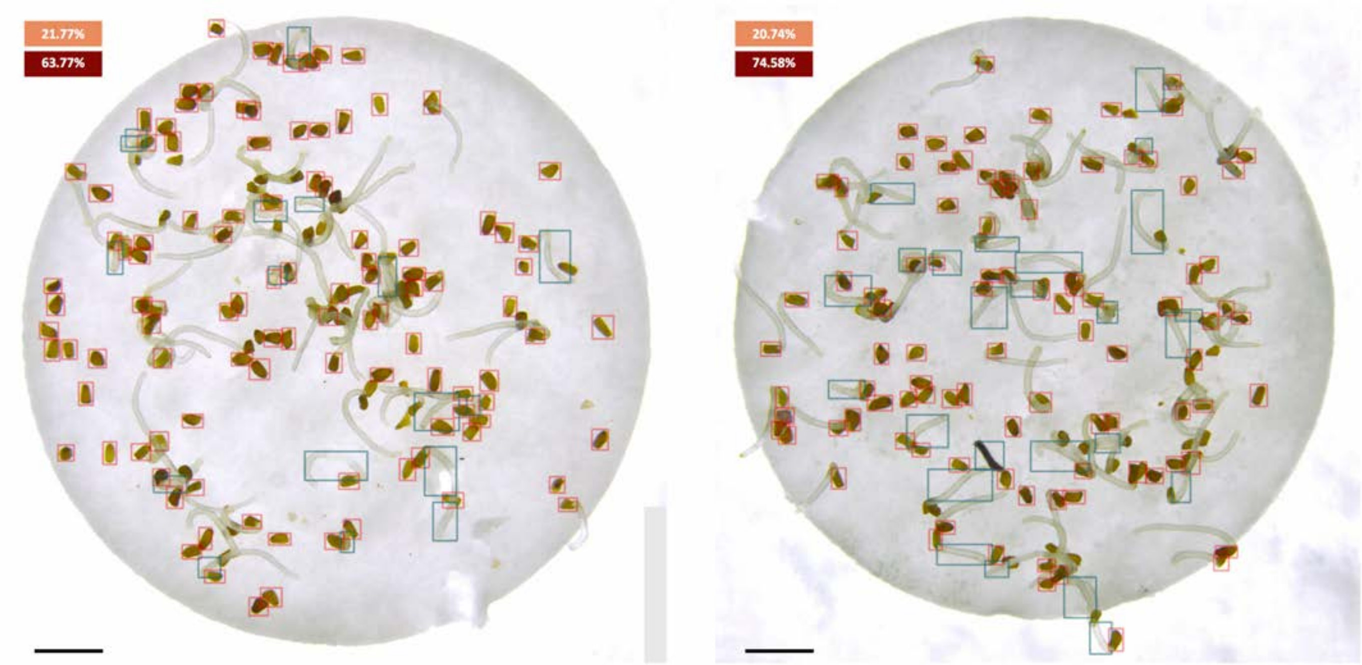

Supplemental Figure S6: R-50-C4 backbone test on long radicles-containing images with *Striga* seeds. To test the limitation of the detection and counting performances of the R-50-C4 backbone following the S/R annotation approach, images containing long radicles (germination time >24 hours) were processed by SeedQuant.

The error percentage for each image is reported in the upper left-hand corner: for the counting of seeds (light red) and radicles (dark red) between the R-50-C4 backbone and the ground truth. The detected seeds are indicated by red bounding boxes, and the radicles by green bounding boxes. The scale bars represent 1cm.

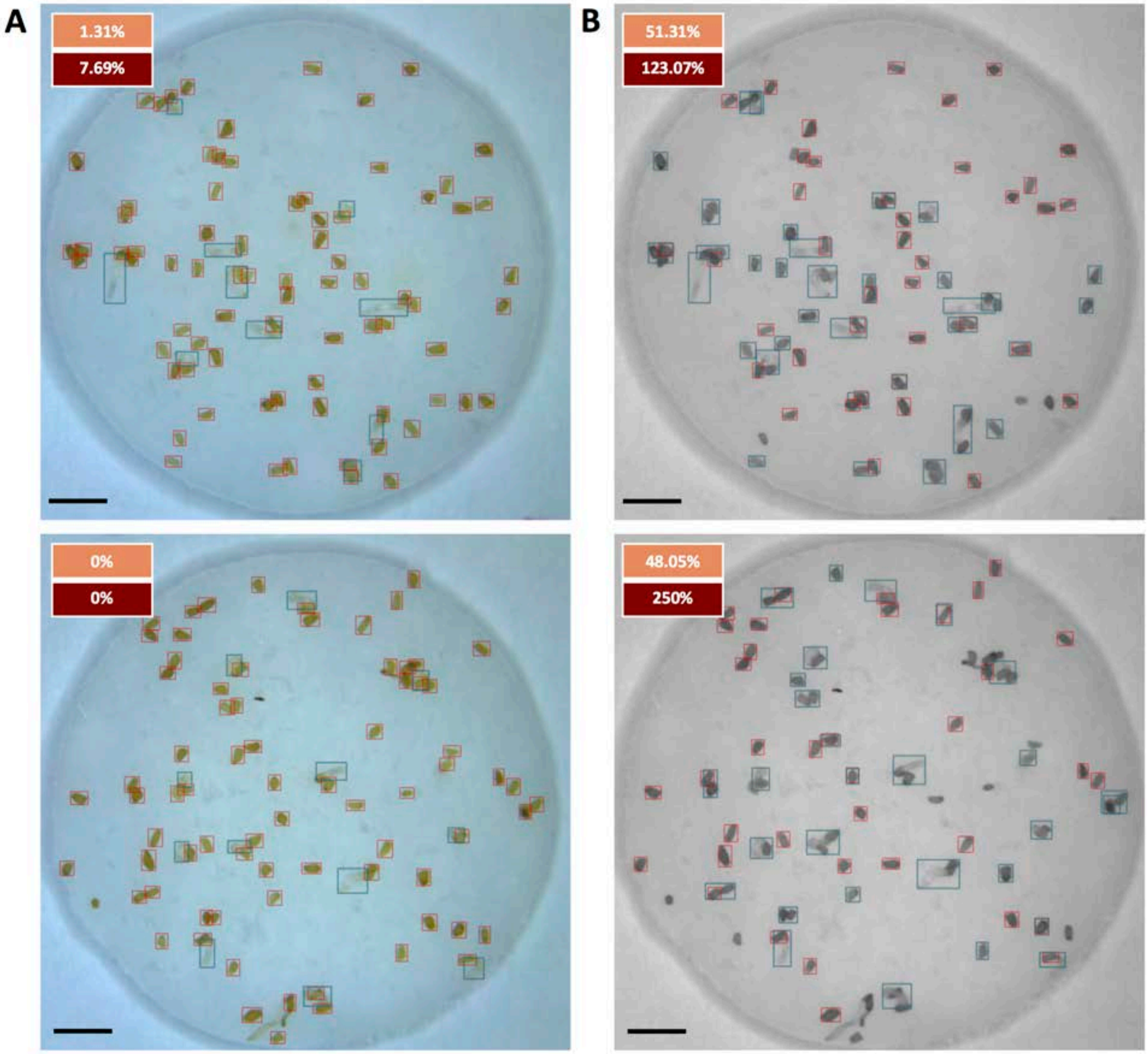

Supplemental Figure S7: R-50-C4 backbone test on grayscale images. To test the color dependence of the detection and counting performances of the R-50-C4 backbone following the S/R annotation approach, images containing **A-** *Striga* seeds were converted to **B-** grayscale and processed by SeedQuant.

The error percentage for each image is reported in the upper left-hand corner: for the counting of seeds (light red) and radicles (dark red) between the R-50-C4 backbone and the ground truth. The detected seeds are indicated by red bounding boxes, and the radicles by green bounding boxes. The scale bars represent 1cm.

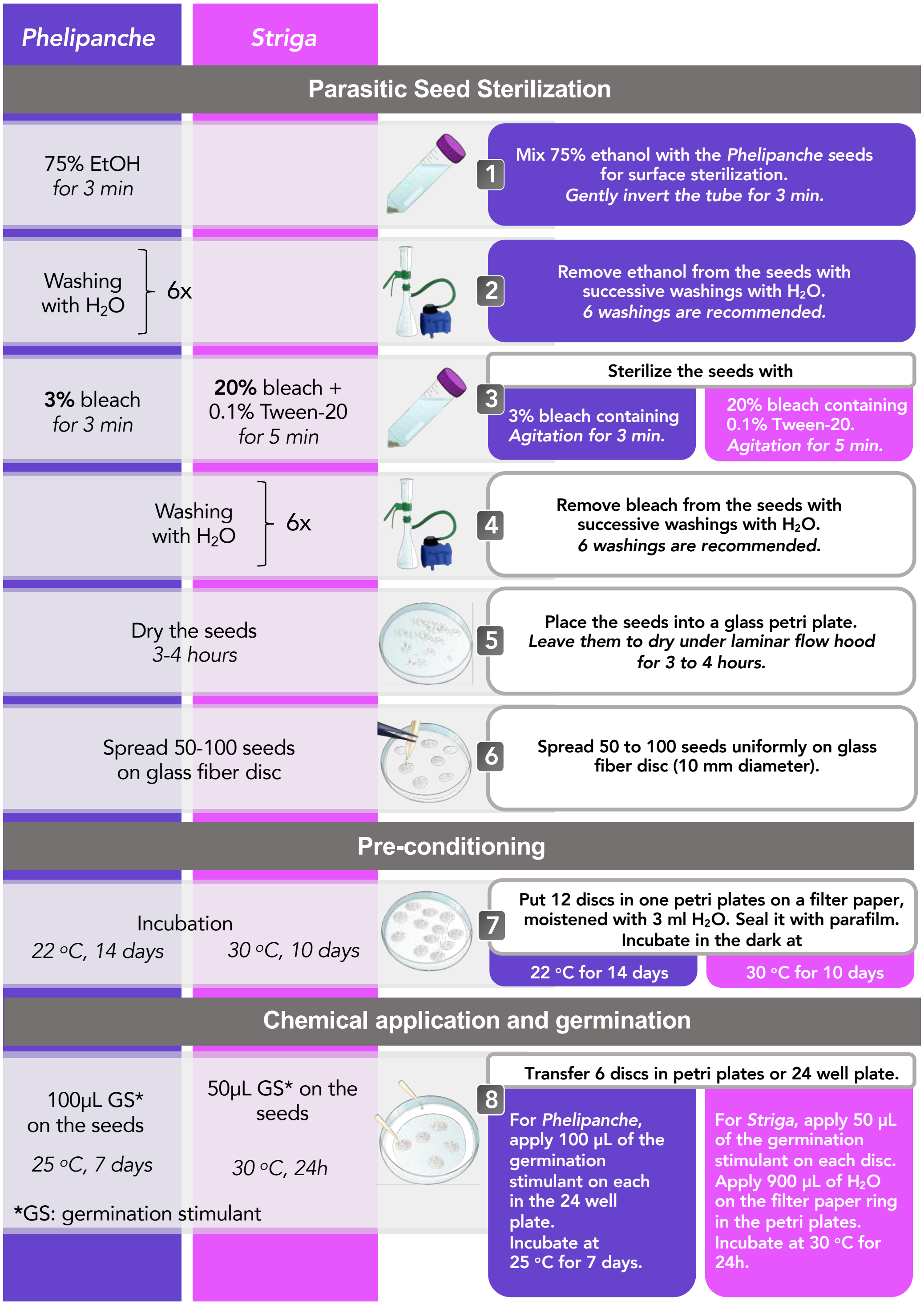

Supplemental Figure S8: Illustrated step-by-step protocol for parasitic seeds bioassay
